# Supplementary material for: Finding the ‘sweet spot’ between customisation and workflows when optimising ePrescribing systems: a multisite qualitative study
Source: BMJ Open. 2022 Dec 5;12(12):e062391. doi: 10.1136/bmjopen-2022-062391 (PMC9723895; doi:10.1136/bmjopen-2022-062391)
Supplement: Supplementary data [file bmjopen-2022-062391supp001.pdf]

Supplementary tables

Supplementary Table 1. Site Selection Criteria

Criterion	Examples
**Significant post implementation experience of ePrescribing system**	HIMMS/Digitally mature (years since EHR implementation)
**Available points of comparison for health system to NHS**	OECD country
**EHR system**	Large integrated systems and Best of Breed
**Vendor**	A mixture of home grown and commercial off the shelf package providers
**Innovative approach**	For example, integrating genomics and other biomedical data, big data feedback into ePrescribing
**Prior interaction with the site/named contact?**	Gatekeeper/<br>network

Supplementary Table 2. eP Opt Study site characteristics

	Hospital details			Participant details			
Site identifier	Location	Size	Type	Roles included in sample	Total number	Vendor or home-grown	Integrated or best of breed (BoB)
Site 1	UK	~760 beds	Teaching hospital	Pharmacy managers,<br>analysts, pharmacists, nurses, information officers	6	Vendor	BoB

Site 2	UK	~800 beds	Teaching hospital	Pharmacy managers, physicians, analysts, pharmacist,<br>Nurses,<br>Other Ancillary care	13	Vendor	Integrated
Site 3	Netherlands	953 beds	Teaching hospital	Clinical pharmacist, nurses, Chief clinical information officer	5	Vendor	Integrated
Site 4	Norway	1,870 beds	Teaching hospital	Pharmacy, physician, nurse, central health I.T clinician	5		
Site 5	US	~80 beds	Paediatric Cancer hospital	Pharmacy managers, physicians, analysts, information officers	9	Vendor	Integrated
Site 6	US	~800 beds	Teaching hospital	Pharmacy managers, physicians, analysts, pharmacists	8	Vendor	Integrated
Site 7	US	~670 beds	Teaching hospital	Physicians,<br>nurses	3	Home-grown	BoB
Site 8	US	~1500 beds	Teaching hospital	Pharmacy managers, physicians, pharmacists<br>Information officers	5	Vendor	Integrated
Site 9	US	~20,000 *	Healthcare Provider	Informatics and	2	Home grown	Integrated

				pharmacy leads			

Supplementary table 3. Quotations by theme.

Quotation number in main manuscript	Interviewee Pseudonym	Quotation
**Safety and workarounds**		
Quotation 2	Site B, pharmacist safety– UK	"Even though they've been taught a standardised route early on they don't remember that. And if you find another way you can find what they believe is a shortcut to do something, but often then some of the safety functions aren't on it because they've gone in a different way."
Quotation 3	Site C, CMIO – Netherlands	*"I mean when I was a CMIO one of the nurses from the gynaecology department, they came to me and they said, hey, we have a problem with Epic, we cannot prescribe medication any more for our ambulatory patients. I said, well, you're not allowed to, legally you're not allowed to prescribe. Yes, but we always did. Yeah, okay, but that was against the law then. Yes, but how can we work that? I said, and how did you do it then, because you had to sign for it? Oh, yes, but we simply always had a blank book of signed prescriptions, they were blank and we just filled in what was needed."*
**Evolution away from highly configurable and customisable solutions**		
Quotation 5	Site F, ambulatory care doctor– US	*"Lot of what we have is customised to us. Some would argue that it's over-customised in that over-customisation you make things so complex that it's hard to like…they become very difficult to work with and very cumbersome".*
Quotation 6	Site E, CDS officer– US	*"Then, it usually gets reviewed and if it's the medication safety group especially, and if it's something we feel like might repeat itself and the rules system can't handle it then we*

		take it through that process of, should we do this, should we put the effort into doing that. And, we might respond and create a custom decision support rule to take care of it, if we feel like it might never happen again, we might not or it's just so complicated that you can't prevent it.."
**Vendor-client relationship**		
Quotation 8	Site B, division pharmacist– UK	"I suppose it depends as well a lot of the functionality we can develop, so the [COTS 1] we've got is in lots of other sites. It's different in all of the sites because people tailor it to what they believe are their own needs... And then if you tailor it so much then you need to be able to deliver all, you almost take more responsibility for your training and everything."
Quotation 9	Site D, IT nurse- Norway	" I did work with the doctors in that part of the project, so we did go around, talk to the doctors, what do they need to learn more about, and what was the frustration, and that we work around that and make new syst…learned how to work smarter for the doctor. So I did go out on the wards and talk to the doctors in their meetings."
**The role of governance**		
Quotation 10	Site H, pharmacy manager– US	*"So, it's important to have a structure, right. On the pharmacy side, we have a few different committees. We have an adult clinical committee, we have a paediatric committee, we have an oncology committee. So, any drug that we want to configure or optimise or modify really needs to be presented to this committee for ultimate approval. And we have a higher- level governance too."*
Quotation 11	Site E, CDS officer– US	*"…the …medication safety resident [has] taken that governance to a little bit of a higher level. Where, he's developed a group of physicians, advanced practice providers, pharmacists, IT professionals that review all those*

		decisions/rules that we have that affect medications and get them to prioritise things. Also, puts data in front of them in how often things are firing, asks for voting on whether or not, you know, a specific piece of decision support should be turned off or enhanced or whatever"
Quotation 12	Site I, pharmacy informatics– US	*"So, we have the ability to check drugs, allergy interactions, across different facilities, so it's called a remote data order check…. …So, we had, we had quite a few instances where patients had a recorded something up, another drug or drug allergy and there are clinical decisions for it, but the electronic medication ordering did not trigger. Why didn't it? And then we were able to say well, the feature was turned off, and now we actually monitor within a day, so if someone turns that feature off today we would know by tomorrow morning and contact them turning it back on again."*
Quotation 13	*Site B, pharmacist safety– UK*	*"I think our electronic prescribing system was chosen because it links into the results and the patient record, so it's one solution, which is brilliant. But then some of the functionality from a prescribing and administration point of view is not there, so therefore that creates risks that we have to then look at… So, if the doctor then says, do you know what, I am going to give it anyway, it doesn't fire an alert for the nurse as they go to administer it; whereas other systems do do that. So, that is an accepted risk with this system. So, we then have to put other systems in place to make staff aware of that and to support that functionality."*
**Finding the 'sweet spot'**		
Quotation 15	*Site I, pharmacy informatics– US*	*"So they would take a facility's innovation and then distribute it to all of the organisation so that it's no*

		longer a customisation, now it's just a feature."
Quotation 16	*Site A, CIO – UK*	*"So really all the time you're trying to pay for a bit of a sweet spot in terms of taking out of the box functionality and configuring it. Is probably a way of describing it. where you're not fundamentally changing the product, but if you can put in configurations that are informed again from the point of view of what has kind of a broad applicability across your profession, and not too specific, tends to give good results."*
