# Supplementary material for: Finding the ‘sweet spot’ between customisation and workflows when optimising ePrescribing systems: a multisite qualitative study
Source: BMJ Open. 2022 Dec 5;12(12):e062391. doi: 10.1136/bmjopen-2022-062391 (PMC9723895; doi:10.1136/bmjopen-2022-062391)
Supplement: Author's manuscript [file bmjopen-2022-062391.draft_revisions.pdf]

BMJ Open is committed to open peer review. As part of this commitment we make the peer review history of every article we publish publicly available.

When an article is published we post the peer reviewers' comments and the authors' responses online. We also post the versions of the paper that were used during peer review. These are the versions that the peer review comments apply to.

The versions of the paper that follow are the versions that were submitted during the peer review process. They are not the versions of record or the final published versions. They should not be cited or distributed as the published version of this manuscript.

BMJ Open is an open access journal and the full, final, typeset and author-corrected version of record of the manuscript is available on our site with no access controls, subscription charges or pay-per-view fees (http://bmjopen.bmj.com).

If you have any questions on BMJ Open's open peer review process please email info.bmjopen@bmj.com

BMJ Open

Finding the 'sweet spot' between customisation and workflows when optimising ePrescribing systems: A multisite qualitative study

Journal:	*BMJ Open*
Manuscript ID	bmjopen-2022-062391
Article Type:	Original research
Date Submitted by the Author:	24-Jun-2022
Complete List of Authors:	Heeney, Catherine; The University of Edinburgh Usher Institute of Population Health Sciences and Informatics<br>Malden, Stephen; The University of Edinburgh Usher Institute of Population Health Sciences and Informatics, Advanced Care Research Centre<br>Sheikh, Aziz; The University of Edinburgh Usher Institute of Population Health Sciences and Informatics, Division of Community Health Sciences
Keywords:	Health informatics < BIOTECHNOLOGY & BIOINFORMATICS, QUALITATIVE RESEARCH, PUBLIC HEALTH

SCHOLARONE™
Manuscripts

BMJ

I, the Submitting Author has the right to grant and does grant on behalf of all authors of the Work (as defined in the below author licence), an exclusive licence and/or a non-exclusive licence for contributions from authors who are: i) UK Crown employees; ii) where BMJ has agreed a CC-BY licence shall apply, and/or iii) in accordance with the terms applicable for US Federal Government officers or employees acting as part of their official duties; on a worldwide, perpetual, irrevocable, royalty-free basis to BMJ Publishing Group Ltd ("BMJ") its licensees and where the relevant Journal is co-owned by BMJ to the co-owners of the Journal, to publish the Work in this journal and any other BMJ products and to exploit all rights, as set out in our licence.

The Submitting Author accepts and understands that any supply made under these terms is made by BMJ to the Submitting Author unless you are acting as an employee on behalf of your employer or a postgraduate student of an affiliated institution which is paying any applicable article publishing charge ("APC") for Open Access articles. Where the Submitting Author wishes to make the Work available on an Open Access basis (and intends to pay the relevant APC), the terms of reuse of such Open Access shall be governed by a Creative Commons licence – details of these licences and which Creative Commons licence will apply to this Work are set out in our licence referred to above.

Other than as permitted in any relevant BMJ Author's Self Archiving Policies, I confirm this Work has not been accepted for publication elsewhere, is not being considered for publication elsewhere and does not duplicate material already published. I confirm all authors consent to publication of this Work and authorise the granting of this licence.

For peer review only

Title: Finding the 'sweet spot' between customisation and workflows when optimising ePrescribing systems: A multisite qualitative study

Authors:

Catherine Heeney1, Stephen Malden1,2 (corresponding author), Aziz Sheikh1

Author affiliations:

Corresponding Author email and address:

Dr Stephen Malden

Email: stephen.malden@ed.ac.uk

1. Centre for Medical Informatics

The Usher Institute

The University of Edinburgh

Old Medical School

Teviot Place

Edinburgh

EH8 9AG

2. Usher Institute | Advanced Care Research Centre (ACRC)

Dr Stephen Malden

Usher Institute | Advanced Care Research Centre (ACRC)

University of Edinburgh, Biocubes, Edinburgh BioQuarter

9 Little France Road, Edinburgh, EH16 4UX

Word count: (2999)

Funding: National Institute for Health Research (NIHR) (Optimising ePrescribing in Hospitals (PR-ST-01–10001)/Policy Research Programme).

For peer review only - http://bmjopen.bmj.com/site/about/guidelines.xhtml

Abstract

Objectives: The introduction of ePrescribing systems offers the potential to improve the safety, quality and efficiency of prescribing, medication management decisions and patient care. However, an ePrescribing system will require some customisation and configuration to capture a range of workflows in particular hospital settings. This can be part of an optimisation strategy, which aims at avoiding workarounds that lessen anticipated safety and efficiency benefits. This paper aims to identify ePrescribing optimisation strategies that can be translated into hospitals in different national settings. We will explore the views of professionals of the impact of configuration and customisation on workflow

Design: This paper draws on 54 qualitative interviews with clinicians, pharmacists and informatics professionals with experience of optimising ePrescribing systems in eight hospital sites and one health system, in four different countries.

Results: Optimisation of ePrescribing systems can involve configuration and/or customisation. This can be a strategy to combat workarounds and to respond to local policy, safety protocols and workflows for particular patient populations. However, it can result in sites taking on responsibility for training and missing out on vendor updates. Working closely with vendors and other users can mitigate the need for extensive system modification and produce better outcomes.

Conclusions: Modifying an ePrescribing system remains key to enhance patient safety and better capture workflow remains key to optimisation. However, we found evidence of an increasingly cautious approach to both customisation and configuration amongst system users. This has lead to users seeking to make less changes to the system.

'Strengths and limitations of this study'

- Whilst there is now a body of work on the implementation of ePrescribing systems the processes of optimisation needed to attain potential benefits is still relatively under explored.
- The study, provides relevant lessons from digitally advanced hospitals across different geographical contexts to be interpreted relevant in relation to ePrescribing systems optimisation in national health systems at scale.
- We focus only on OECD countries this means that we will have failed to capture interesting examples of ePrescribing beyond that.
- Some types of system are over represented, in particular integrated systems provided by EPIC an Cerner were present in the majority of our sites.

Funding:

This study/project is funded by the National Institute for Health Research (NIHR) (Optimising ePrescribing in Hospitals (PR-ST-01–10001)/Policy Research Programme). The views expressed are those of the author(s) and not necessarily those of the NIHR or the Department of Health and Social Care.

For peer review only - http://bmjopen.bmj.com/site/about/guidelines.xhtml

Introduction

Around ten per cent of preventable harms to inpatients is attributable to errors in the prescribing process (1). Electronic health record (EHR) systems offer the potential to improve the safety of prescribing and medication management decisions and patient care (2). The introduction of such systems have the potential to minimise medication error (3). However, it is also acknowledged that an EHR will not be perfectly adapted to capture existing workflows in a given hospital and will require fine tuning to local safety protocols as well as professional and specialisation needs (4, 5). Developing the ePrescribing system in situ is key to making it safe to use, in a 'high risk' setting such as health care (6). In the complex and evolving secondary care context it is unrealistic to imagine that commercial off-the-shelf (COTS) systems can offer optimal performance with local or indeed wider organisational requirements immediately post-implementation (7, 8).

Changes to an ePrescribing system can take the form of configuration and customisation. Configuration works with existing options available in the system or from the vendor by changing and refining rules to reflect processes and practices in local and national settings (7). Customisation involves more fundamental coding changes or 'modifying the underlying software to improve functionality' (9). Hospitals resort to this where the vendor does not provide sufficient configurability to capture necessary workflows (10).

Workarounds arise where there is a mismatch between practices within particular hospitals and the functionalities and capacities of the ePrescribing system [5(4, 11)] (12-14). They remain a problem especially when they by pass in built safety features [6[7(1)]. Configuration and customisation may look like an obvious win in terms of better capturing workflow. Moreover, this route can be

For peer review only - http://bmjopen.bmj.com/site/about/guidelines.xhtml

necessary where, for example, there are very particular patient populations or national policy requirements (10). However, drawbacks of extensive configuration and customisation include being left behind for standard updates from the vendor and uncertainty as to where responsibility for making improvements resides (9, 10).

Modifications to reflect to mediate local requirements and system capabilities are key to system optimisation (4, 7, 15), which has been defined as, 'the activity of enhancing system capabilities and integration of subsystem elements to the extent that all components operate at or above user expectations' (16). Hospitals face an inevitable tension between an objective to offer a universal, coordinated and standardised approach to system functionality on the part of the vendor and national health systems, and a requirement to accommodate specific workflows at local level [3, 5(10)]. The impact and learning arising from optimisation strategies, reliant on configuration and customisation, over time is still relatively unexplored in existing literature on ePrescribing. Below, we will discuss strategies used to incorporate and manage ePrescribing systems and workflow and the mutual process of change that this interaction produces as well as shifts to in attitudes to modifying system functionality.

Methodology

The qualitative fieldwork described in this paper is part of a wider study on Optimisation of ePrescribing in Hospitals (eP Opt). The methods employed for this present study are outlined below, for a more detailed description see (17). We employed a qualitative multi-site study design with semi-structured interviews carried out in each of the study sites. The aim was to capture strategies and practices for optimisation of ePrescribing in particular hospitals, using different systems and dealing with diverse national infrastructure and policy.

For peer review only - http://bmjopen.bmj.com/site/about/guidelines.xhtml

Selection of study sites:

Eight hospitals and one health care provider participated in this study across four countries (US, UK, Netherlands and Norway). All sites had significant experience of implementation and optimisation of EHRs and ePrescribing, Computerised Physician Order Entry (CPOE) and Clinical Decision Support (CDS). All hospitals had begun digitisation at some level at least a decade before fieldwork began. We selected sites hospitals awarded Healthcare Information and Management Systems Society (HIMSS) level 6 or 7, HIMMS is a widely used measure of digital excellence. The eight hospitals were high profile teaching hospitals. A purposive sampling strategy was used to select cases (18) (see (Supplementary Table 1). We identified sites through a scoping review of optimisation strategies in ePrescribing (19) and two expert roundtable events (17). Only Organisation for Economic Cooperation and Development (OECD) were included to increase the internal comparability of the sample.

(Supplementary table 1)

Data collection:

We contacted 10 hospitals in total. In all cases, we had an initial meeting with a gatekeeper(s) who helped identify relevant professionals. Due to COVID restrictions and lack of staff availability one site in Spain, did not take part. In the remaining sites (see supplementary Table 1), we contacted relevant professionals by email, with a consent form and information sheet. We interviewed 54 professionals including clinicians, Chief Information Officers (CIOs), Chief Medical Information Officers (CMIOs), pharmacists and I.T and data specialists [7].

For peer review only - http://bmjopen.bmj.com/site/about/guidelines.xhtml

For peer review only

Planned site visits were replaced by remote interviews using approved online platforms, including Teams, Zoom, nhn.no and Skype. Initial contact with the sites was in early 2020, with interviews beginning in the first site in May 2020 and the final interview was conducted in May 2021. Two experienced qualitative researchers (CH and SM) conducted semi-structured interviews following an interview topic guide (supplementary material). The researchers had no prior relationship with participants. Interviews lasted between 30 and 90 minutes depending on the interviewees' time and availability and were recorded and transcribed verbatim.

Data analysis

The research team (CH and SM) first independently coded two transcripts and discussed any discrepancies before finalising the coding framework (supplementary material). The researchers then coded all 54 transcripts. Transcripts were analysed using an inductive thematic analysis and the data grouped into themes and sub-themes (20). We employed NVivo 12 pro qualitative data analysis software. For this paper, we extracted data coded to relevant codes including configuration and workflow, which were then further categorised into five cross cutting themes.

Results

Drawing on 54 semi-structured interviews across nine different sites, in four countries (see supplementary Table 2). We identified five themes, influencing both workarounds and configuration:

For peer review only - http://bmjopen.bmj.com/site/about/guidelines.xhtml

For peer review only

safety and workarounds, evolution away from highly configurable and customisable solutions, vendor-client relationship, the role of governance and *finding the 'sweet spot'*.

Six of our fieldwork sites had opted to purchase an integrated commercial system, rather than maintaining their own home grown system. Although two sites still employed the so called 'Best of Breed' model (see supplementary Table 2).

(Supplementary table 2.)

Safety and workarounds

As previous work has acknowledged changes to the system are in many cases needed to allow functionality, which enables particular workflows. Changes were needed to reflect workflows beyond the North American context where the system had been developed. Barcode scanning of medicines, which is increasingly a safety feature included in many commercial systems, did not always match the labelling of available products with clear safety, which meant staff created a workaround.

(see quotation 1 *Table 3: supplementary material*)

Individuals improvised particular processes when they were not sure how to follow the 'standardised route'.

For peer review only

(see quotation 2 *Table 3: supplementary material)*

In some cases, staff struggled to adjust when the system curtailed workarounds. Whilst this increased safety, it meant staff could no longer resort to shortcuts to save time.

(see quotation 3 *Table 3: supplementary material)*

Evolution away from highly configurable and customisable solutions

A number of participants noted the drawbacks of a highly modifiable and flexible systems, whilst accepting the benefits of taking are more cautious approach to modification. Several sites described how vendors had supported a high degree of local customisation or configuration in the early days of implementation. This was often the case when the vendor was trying to roll out their system in a new national or speciality context.

(see quotation 4 Table 3: supplementary material)

It later became clear to a number of sites as well as vendors that too much modification could lead to an unwieldy system creating extra work for the vendor.

(see quotation 5 Table 3: Supplementary material)

For peer review only - http://bmjopen.bmj.com/site/about/guidelines.xhtml

Whilst the system now has the functionality to mimic the ‘traditional drug chart view’, this was considered less valuable than the speed and usability for all clinical areas, to which users had become accustomed. Those with responsibility for modifying the system need to balance the safety risks arising from a lack of system functionality with potential over customisation. Experience of the specific needs of a particular site coupled with familiarity with a vendor mean that staff are able to make more informed judgements about whether taking a configuration route was the best way to improve safety overall.

(see quotation 6 Table 3: supplementary material)

Vendor-client relationship

Several interviewees noted that making highly specific modifications of the system risk opening a gulf between the site and the vendor. Whilst there is a view that sites are clients and have some rights to ask for what they want, a number of interviewees acknowledged that suppliers provide a service at a general level and that the sites can feed into that.

(see quotation 7 Table 3: supplementary material)

Tailoring the system to a very high degree can also lead to individual sites having to take responsibility for ensuring that staff understand and can safely access the specific customised or configured functionalities.

For peer review only - http://bmjopen.bmj.com/site/about/guidelines.xhtml

(see quotation 8 Table 3: supplementary material)

A mutual shaping of needs and vision was occurring in a number of sites, where key individuals had developed close working relationships with the vendors. Those staff selected to complete extensive training pre-implementation would then move on to be 'super users'. This was a mechanism was used as a strategy to bring existing workflows together with the system's capacities to avoid both workarounds and excessive customisation.

(see quotation 9 Table 3: supplementary material)

The role of governance

Many interviewees balanced a recognition that changes should be minimal with an acceptance that vendors cannot design systems to fit every context. This meant that governance and monitoring formed a huge part of how staff prioritised and retained particular configurations or customisations.

(see quotation 10 Table 3: supplementary material)

Internal governance is necessary to consider both petitions for customisation before they are forwarded to the vendor and to prioritise configurations in relation to, for example, alert functions or protocols for specific processes. A data led approach, enabled by system functionality, can be

For peer review only - http://bmjopen.bmj.com/site/about/guidelines.xhtml

used to inform meetings with relevant professionals regarding prioritisation of particular configurations of the system.

(see quotation 11 Table 3: supplementary material)

An interviewee with oversight of a number different hospital sites run by a single health provider, monitored and reversed configurations to, for example alert systems, in order to enforce and support uniform expectations about safety functions.

(see quotation 12 Table 3: supplementary material)

Simultaneously, data on any alert rule changes in local hospital sites was reviewed as a basis for prioritising those changes, which were useful at the local level. Staff also need to work towards an awareness that the system at any given time will have limitations and may not be doing everything that people think it does.

(see quotation 13 Table 3: supplementary material)

Finding the 'sweet spot'

Vendors can encourage greater conformity by ensuring that only technologies and practices that follow the rules can take advantage of the interoperability and integration opportunities offered by

For peer review only - http://bmjopen.bmj.com/site/about/guidelines.xhtml

their products. One US based CMIO made a comparison to the technology company Apple, which limits how its products are modified or used with other products.

(see quotation 14 Table 3: supplementary material)

In this case, the interviewee did not see the imposition of standards as negative but rather as contributing to a more 'consistent' experience for users.

(see quotation 15 Table 3: supplementary material)

One strategy, explicitly encouraged by vendors in some cases, was for individual sites to have their needs met as part of a network of users. Similarly, in the following instance, where the site formed part of a larger health care organisation with one shared system, the was an emphasis placed on only pushing for those changes that could be enacted in every site.

(see quotation 16 Table 3: supplementary material)

The ability to manage the tension between local and wider applicability of a system modification, was referred to as finding 'a sweet spot' between 'out of the box functionality and configuring it.'

Discussion

For peer review only

For peer review only - http://bmjopen.bmj.com/site/about/guidelines.xhtml

This qualitative study involving digitally advanced, hospitals highlights a number of important principles around customisation and configuration to be considered when optimising an ePrescribing system. Misalignment of functionality and organisational needs is seen as one reason why hospitals go down the customisation route (4). Our data suggests that EPIC and Cerner, which were designed in a North American context required changes to functionality to capture workflows in other national settings. The differences in roles, workflows and policy imperatives for access to particular data sources in the different countries required an early rethink soon after implementation. Where sites dealt directly with the vendor, the hospital worked with the vendor to explain and introduce particular national requirements.

Workarounds continue to be a complex issue that require substantial consultation with providers. Workarounds may allow staff and systems to gradually coalesce [7] whilst avoiding the pitfalls of over customisation [19]. We found cautious approach to configuration and customisation was accompanied by low tolerance for the long term use of workarounds. There was a high degree of awareness about the potential safety and administrative drawbacks of by passing the system. Staff had also developed vigilance with regard to the limitations of the system and they need for caution about what safety functions are actually enabled at any given time.

The mismatch between workflow and system functionality has been combatted in a number of sites by so called super users who are deployed to encourage staff to work within the systems capacities. Sites had learned over time to be more adaptable in accepting the way that the balance between their own and vendors' needs. In some cases, key staff members developed a close relationship with the vendor, allowing them to maintain a current appreciation of the potentialities of the system.

For peer review only - http://bmjopen.bmj.com/site/about/guidelines.xhtml

Innovative forms of monitoring and testing the effectiveness of particular configurations were in evidence, with one site in particular employing data to monitor acceptance and use of particular alerts. This data driven approach was combined with staff oversight and comparison with the uptake of similar alerts across other sites. This allowed rigorous monitoring and prioritisation work to balance beneficial and helpful optimisation against the drawbacks of making multiple changes to system. Drawbacks included increasing responsibility on the sites for training, maintenance and improvement.

Finding the 'sweet spot' appeared to involve a subtle evolution away from a demand for highly configurable and customisable solutions and towards finding solutions that could be up scaled. Sites were apt to search for allies within the national system user network to lobby for or finance requested changes to the system. Therefore, in order to fix a mismatch between functionality and workflow sites would need to be proactive in finding other users experiencing similar problems. There appeared to be an increasing willingness for sites to encourage behaviour change in staff and to scale back their requests for changes to the system driven by both safety and usability concerns.

Strengths and limitations

This is one of the first studies to investigate the impact of ePrescribing optimisation attained by customisation and configuration practices. The case studies presented provide insight into diverse regional and national contexts. The focus on optimisation enables the sharing of key learning in the interactive evolution of users and system. By focussing on professionals within advanced sites, we have been able to capture the benefits of their experiences post implementation.

For peer review only - http://bmjopen.bmj.com/site/about/guidelines.xhtml

For peer review only

Due in part to the disruption caused during fieldwork by COVID-9, we did not collect equal levels of data in all sites. This was mitigated wherever possible by our carrying out longer interviews. Hospitals using two integrated systems or COTS (Epic and Cerner) are dominant within the sample, best-of-breed systems are far less represented. The US and UK contexts are over represented compared to the Netherlands and Norway. The focus on OECD member countries narrowed the range of hospitals we could include.

Interpretation in the light of the wider published literature

Customisation has been presented as giving positive outcomes for users and patients (21). It is also inevitable given the difficulty of capturing workflows without knowledge of the environment and actual challenges faced by staff (8). Whilst the system may be designed to reshape practices in a more efficient and safer ways, initially there is often misalignment between what is required by the specialisation or organisation and the functionality of the system (4, 10). Vendors worked closely with early adopters bringing a lot of specialised knowledge about national settings and specialities to the relationship, which our data suggests enabled customisation. This appeared to become less desirable not only to vendors but more recently also to users, over time. Hospital sites are increasingly willing to adopt a parsimonious approach to configuration and customisation.

The mutual learning between site and vendor is an ongoing process requiring staff and resourcing. Where systems are developed in a distinct national context a network of other users may be sought out in developing necessary functionality at a quicker pace. More recent studies have pointed to greater awareness of the balance of costs and benefits in relation to extensive modifications, including wide variation between sites using the same system (4, 22). Future research could look at

For peer review only

the ways in which relationships between vendor and individual sites are shaped by the presence of a critical mass of users in a given national context.

Conclusion

Our data suggests an increasing acceptance for interviewees that the needs of individual sites would be met as part of a network of users potentially of the same product. Whilst some frustrations with delays on the part of the vendor to changes required by sites remained, there was not much enthusiasm for making too many changes at a local level. Sites acknowledged the danger of becoming too responsible for an extremely bespoke system. Simultaneously, interviewees were cognisant that the vendor would not foresee all eventualities, especially in specialities or within scientifically as well as digitally advanced hospitals. However, they had learned the benefit of considering broad applicability of optimisations.

Contributors

AS conceptualised the project and designed the study. CH recruited study sites; CH and SM recruited individual participants within study sites and conducted data collection. CH prepared the first draft of the manuscript. All authors contributed to the final draft of the manuscript and approved it for submission.

Competing interests:

None

Patient and public involvement:

For peer review only - http://bmjopen.bmj.com/site/about/guidelines.xhtml

Patients and/or the public were involved in the design or conduct or reporting or dissemination plans of this research.

Ethics approval:

This study was granted ethical approval by the Usher Research Ethics Group (University of Edinburgh) on 21/01/2020 (ref.1906), and the relevant NHS research and development approvals were acquired for UK-based sites on 23/01/2020 (ref.19/HRA/7015). As this research is exclusively being conducted with healthcare professionals, there is no involvement of vulnerable groups. As we are focusing this research on well-known, world-leading institutions in health informatics, extra care should be taken when presenting research findings to not compromise the anonymity of the respondents. Specific job titles have been masked when used in conjunction with individual quotes, and care has been taken to avoid any quotes that could be used to potentially identify individuals. All audio files, transcripts and consent forms will be kept in a secure, password-protected folder within the University of Edinburgh's DataStore (for further information please consult the protocol for this study (17)).

Acknowledgments:

We thank all the participating sites and the individuals who participated in an interview for this study. We thank Serena Tricarico, Kieran Turner, Toni Wigglesworth, and our Patient and Public Involvement representatives, Antony Chuter and Jillian Beggs, for their support and feedback throughout the project. We also acknowledge the support of colleagues from the Department of Health and Social Care, the National Health Service and the Medicines and Healthcare products Regulatory Agency: Ann Slee (NHS), Jason Cox (DHSC), Richard Cattell (NHS), Helen Causley (DHSC), Paul Stonebrook (DHSC), Mick Foy (MHRA), Kathryn Ord (MHRA), and Graeme Kirkpatrick (NHS). We thank the referees for reviewing this manuscript.

For peer review only - http://bmjopen.bmj.com/site/about/guidelines.xhtml

For peer review only

Patient and public involvement

A major component of the eP Opt project is the involvement of patient and public representatives across the four project phases. Specifically, two patient and public (PPI) representatives are involved as team members, who attend research meetings and public events to provide feedback and suggestions on the work within each phase from a patient's perspective. They have also been extensively involved with assisting with the design of an upcoming PPI roundtable event for the project, progress of the study has been shared with a group of invited patients, and their feedback brought to bear on research decisions and directions. These PPI consultations have helped in the formulation of research questions and fed into analysis of the data by highlighting current gaps in practice from a patient perspective.

References

1. Puaar SJ, Franklin BD. Impact of an inpatient electronic prescribing system on prescribing error causation: a qualitative evaluation in an English hospital. BMJ Qual Saf. 2018;27(7):529-38.
2. Cornford T, Dean B, Savage I. et al. Electronic prescribing in hospitals-challenges and lessons learned. 2009.
3. Bates DW, Teich JM, Lee J. et al. The impact of computerized physician order entry on medication error prevention. J Am Med Inform Assoc. 1999;6(4):313-21.
4. Wiegel V, King A, Mozaffar H. et al. A systematic analysis of the optimization of computerized physician order entry and clinical decision support systems: A qualitative study in English hospitals. Health Informatics J. 2019:1460458219868650.
5. Salmon JW, Jiang R. E-prescribing: history, issues, and potentials. Online J Public Health Inform. 2012;4(3).
6. Wachter R. Making IT work: harnessing the power of health information technology to improve care in England. London, UK: Department of Health. 2016.
7. Anderson S, Hardstone G, Procter R. et al. Down in the (data) base (ment): Supporting configuration in organizational information systems. Resources, co-evolution and artifacts: Springer; 2008. p. 221-53.
8. Coiera E. Designing interactions. ROUTLEDGE HEALTH MANAGEMENT SERIES. 2003:97.
9. Sittig DF, Belmont E, Singh H. Improving the safety of health information technology requires shared responsibility: It is time we all step up. Healthcare; 2018: Elsevier.
10. Mozaffar H, Williams R, Cresswell KM. et al. The challenges of implementing packaged hospital electronic prescribing and medicine administration systems in UK hospitals: premature

For peer review only - http://bmjopen.bmj.com/site/about/guidelines.xhtml

purchase of immature solutions? Information Infrastructures within European Health Care. 2017:129-49.
11. Mozaffar H, Cresswell KM, Williams R. et al. Exploring the roots of unintended safety threats associated with the introduction of hospital ePrescribing systems and candidate avoidance and/or mitigation strategies: a qualitative study. BMJ Qual Saf. 2017;26(9):722-33.
12. Boonstra A, Jonker TL, van Offenbeek MA et al. Persisting workarounds in Electronic Health Record System use: types, risks and benefits. BMC Med Inform Decis Mak.2021;21(1):1-14.
13. Cresswell KM, Mozaffar H, Lee L et al. Workarounds to hospital electronic prescribing systems: a qualitative study in English hospitals. BMJ Qual Saf. 2017;26(7):542-51.
14. Ser G, Robertson A, Sheikh A. A qualitative exploration of workarounds related to the implementation of national electronic health records in early adopter mental health hospitals. PloS One. 2014;9(1):e77669.
15. Moon MC, Hills R, Demiris G. Understanding optimisation processes of electronic health records (EHRs) in select leading hospitals: a qualitative study. BMJ Health Care Inform. 2018;25(2).
16. Kidd TT. Handbook of research on technology project management, planning, and operations: IGI Global; 2009.
17. Heeney C, Malden S, Sheikh A. Protocol for a qualitative study to identify strategies to optimise hospital ePrescribing systems. BMJ Open. 2021;11(1):e044622.
18. Patton MQ. Qualitative research & evaluation methods: Integrating theory and practice: Sage publications; 2014.
19. Williams J, Malden S, Heeney C. et al. Optimizing Hospital Electronic Prescribing Systems: A Systematic Scoping Review. Journal of Patient Safety. 2021.
20. Braun V, Clarke V. Using thematic analysis in psychology. Qualitative research in psychology. 2006;3(2):77-101.
21. Adeola M, Azad R, Kassie GM. et al. Multicomponent interventions reduce high-risk medications for delirium in hospitalized older adults. Journal of the American Geriatrics Society. 2018;66(8):1638-45.
22. Pontefract SK, Hodson J, Slee A. et al. Impact of a commercial order entry system on prescribing errors amenable to computerised decision support in the hospital setting: a prospective pre-post study. BMJ Qual Saf. 2018;27(9):725-36.

For peer review only

For peer review only - http://bmjopen.bmj.com/site/about/guidelines.xhtml

Supplementary Table 1. Site Selection Criteria

Criterion	Examples
**Significant post implementation experience of ePrescribing system**	HIMMS/Digitally mature (years since EHR implementation)
**Available points of comparison for health system to NHS**	OECD country
**EHR system**	Large integrated systems and Best of Breed
**Vendor**	A mixture of home grown and commercial off the shelf package providers
**Innovative approach**	For example, integrating genomics and other biomedical data, big data feedback into ePrescribing
**Prior interaction with the site/named contact?**	Gatekeeper/<br>network

For peer review only

Supplementary Table 2. eP Opt Study site characteristics

	Hospital details			Participant details			
Site identifier	Location	Size	Type	Roles included in sample	Total number	Vendor or home-grown	Integrated or best of breed (BoB)
Site 1	UK	~760 beds	Teaching hospital	Pharmacy managers, analysts, pharmacists, nurses, information officers	6	Vendor	BoB
Site 2	UK	~800 beds	Teaching hospital	Pharmacy managers, physicians, analysts, pharmacist, Nurses, Other Ancillary care	13	Vendor	Integrated
Site 3	Netherlands	953 beds	Teaching hospital	Clinical pharmacist, nurses, Chief clinical information officer	5	Vendor	Integrated
Site 4	Norway	1,870 beds	Teaching hospital	Pharmacy, physician, nurse, central health I.T clinician	5		
Site 5	US	~80 beds	Paediatric Cancer hospital	Pharmacy managers, physicians, analysts, information officers	9	Vendor	Integrated
Site 6	US	~800 beds	Teaching hospital	Pharmacy managers, physicians,	8	Vendor	Integrated

For peer review only

				analysts, pharmacists			
Site 7	US	~670 beds	Teaching hospital	Physicians, nurses	3	Home-grown	BoB
Site 8	US	~1500 beds	Teaching hospital	Pharmacy managers, physicians, pharmacists Information officers	5	Vendor	Integrated
Site 9	US	~20,000 *	Healthcare Provider	Informatics and pharmacy leads	2	Home grown	Integrated

For peer review only

Table 3. Quotations by theme

**Safety and workarounds**		
Quotation number in main manuscript	Interviewee Pseudonym	Quotation
Quotation 1	Site C, vendor liaison nurse- Netherlands	"The biggest problem we have is when we can't scan products. In the beginning, we had a lot of troubles with that because as a nurse I scan the [label] but then I scan the antibiotics and the system says hey, I don't know these antibiotics. So I had to make a workaround …There were a lot of wards who had their own work around, and that's something we discovered in the medication commission committee, and we had to work at that, and then we discovered it was due to the barcode scanning"
Quotation 2	Site B, pharmacist safety– UK	"Even though they've been taught a standardised route early on they don't remember that. And if you find another way you can find what they believe is a shortcut to do something, but often then some of the safety functions aren't on it because they've gone in a different way."
Quotation 3	Site C, CMIO – Netherlands	*"I mean when I was a CMIO one of the nurses from the gynaecology department, they came to me and they said, hey, we have a problem with Epic, we cannot prescribe medication any more for our ambulatory patients. I said, well, you're not allowed to, legally you're not allowed to prescribe. Yes, but we always did. Yeah, okay, but that was against the law then. Yes, but how can we work that? I said, and how did you do it then, because you had to sign for it? Oh, yes, but we simply always had a blank book of signed prescriptions, they were blank and we just filled in what was needed."*
**Evolution away from highly configurable and customisable solutions**		

For peer review only

Quotation 4	Site E, CMIO– US	"[COTS 1] very much supported us to, sort of, go out and build things out the way that'd work for us. And a lot of the decisions we made at the very start are things that are coming back and causing problems now…is that we went to every single clinic and asked them to design their own documentation. We made a decision that every single clinical trial would have its own set of orders. And so, now we have 4,500 different power plans associated with clinical trials – chemotherapy plans – and it is impossible to maintain".
Quotation 5	Site F, ambulatory care doctor– US	*"Lot of what we have is customised to us. Some would argue that it's over-customised in that over-customisation you make things so complex that it's hard to like…they become very difficult to work with and very cumbersome".*
Quotation 6	Site E, CDS officer– US	*"Then, it usually gets reviewed and if it's the medication safety group especially, and if it's something we feel like might repeat itself and the rules system can't handle it then we take it through that process of, should we do this, should we put the effort into doing that. And, we might respond and create a custom decision support rule to take care of it, if we feel like it might never happen again, we might not or it's just so complicated that you can't prevent it.."*
**Vendor-client relationship**		
Quotation 7	Site A, CIO – UK	*"I talk about influencing the shape of a product it doesn't necessarily mean that you can ask for a very specific bit of functionality that nobody else wants and you're going to get. But actually, as I say, you do have influence if you're doing interesting things that actually the broader health community are interested in. So you've got to constantly have a mind of the suppliers are out there to run their*

For peer review only

		business and the thing that they're really interested in is maximising the reach of their product. So they really want to talk to you when you're doing things that are actually interesting in a broader context…if you drive customisation to a point where what you're doing is unique, you're setting yourself up to fail.”
Quotation 8	Site B, division pharmacist– UK	“I suppose it depends as well a lot of the functionality we can develop, so the [COTS 1] we've got is in lots of other sites. It's different in all of the sites because people tailor it to what they believe are their own needs... And then if you tailor it so much then you need to be able to deliver all, you almost take more responsibility for your training and everything.”
Quotation 9	Site D, IT nurse- Norway	“ I did work with the doctors in that part of the project, so we did go around, talk to the doctors, what do they need to learn more about, and what was the frustration, and that we work around that and make new syst…learned how to work smarter for the doctor. So I did go out on the wards and talk to the doctors in their meetings.”
**The role of governance**		
Quotation 10	Site H, pharmacy manager– US	*“So, it's important to have a structure, right. On the pharmacy side, we have a few different committees. We have an adult clinical committee, we have a paediatric committee, we have an oncology committee. So, any drug that we want to configure or optimise or modify really needs to be presented to this committee for ultimate approval. And we have a higher- level governance too.”*
Quotation 11	Site E, CDS officer– US	*“…the …medication safety resident [has] taken that governance to a little bit of a higher level. Where, he's developed a group of physicians, advanced practice providers, pharmacists, IT professionals that review all those*

For peer review only

		decisions/rules that we have that affect medications and get them to prioritise things. Also, puts data in front of them in how often things are firing, asks for voting on whether or not, you know, a specific piece of decision support should be turned off or enhanced or whatever"
Quotation 12	Site I, pharmacy informatics– US	*"So, we have the ability to check drugs, allergy interactions, across different facilities, so it's called a remote data order check…. …So, we had, we had quite a few instances where patients had a recorded something up, another drug or drug allergy and there are clinical decisions for it, but the electronic medication ordering did not trigger. Why didn't it? And then we were able to say well, the feature was turned off, and now we actually monitor within a day, so if someone turns that feature off today we would know by tomorrow morning and contact them turning it back on again."*
Quotation 13	*Site B, pharmacist safety– UK*	*"I think our electronic prescribing system was chosen because it links into the results and the patient record, so it's one solution, which is brilliant. But then some of the functionality from a prescribing and administration point of view is not there, so therefore that creates risks that we have to then look at… So, if the doctor then says, do you know what, I am going to give it anyway, it doesn't fire an alert for the nurse as they go to administer it; whereas other systems do do that. So, that is an accepted risk with this system. So, we then have to put other systems in place to make staff aware of that and to support that functionality."*
**Finding the 'sweet spot'**		
Quotation 14	Site H, CMIO– US	*"Apple will say, no, you have to follow our rules, and if your app doesn't follow these rules, we won't*

For peer review only

		let you use it on our platform. The overall user experience is tighter and more consistent. I think, [COTS 2] is a bit like that..."
Quotation 15	*Site I, pharmacy informatics– US*	*"So they would take a facility's innovation and then distribute it to all of the organisation so that it's no longer a customisation, now it's just a feature."*
Quotation 16	*Site A, CIO – UK*	*"So really all the time you're trying to pay for a bit of a sweet spot in terms of taking out of the box functionality and configuring it. Is probably a way of describing it. where you're not fundamentally changing the product, but if you can put in configurations that are informed again from the point of view of what has kind of a broad applicability across your profession, and not too specific, tends to give good results."*

For peer review only

Consolidated criteria for reporting qualitative studies (COREQ): 32-item checklist

Please indicate in which section each item has been reported in your manuscript. If you do not feel an item applies to your manuscript, please enter N/A.

For further information about the COREQ guidelines, please see Tong *et al.*, 2017: https://doi.org/10.1093/intqhc/mzm042

No.	Item	Description	Section #
**Domain 1: Research team and reflexivity**			
Personal characteristics			
1.	Interviewer/facilitator	Which author/s conducted the interview or focus group?	
2.	Credentials	What were the researcher's credentials? *E.g. PhD, MD*	
3.	Occupation	What was their occupation at the time of the study?	
4.	Gender	Was the researcher male or female?	
5.	Experience and training	What experience or training did the researcher have?	
Relationship with participants			
6.	Relationship established	Was a relationship established prior to study commencement?	
7.	Participant knowledge of the interviewer	What did the participants know about the researcher? *E.g. Personal goals, reasons for doing the research*	
8.	Interviewer characteristics	What characteristics were reported about the interviewer/facilitator? *E.g. Bias, assumptions, reasons and interests in the research topic*	
**Domain 2: Study design**			
Theoretical framework			
9.	Methodological orientation and theory	What methodological orientation was stated to underpin the study? *E.g. grounded theory, discourse analysis, ethnography, phenomenology, content analysis*	
Participant selection			
10.	Sampling	How were participants selected? *E.g. purposive, convenience, consecutive, snowball*	
11.	Method of approach	How were participants approached? *E.g. face-to-face, telephone, mail, email*	
12.	Sample size	How many participants were in the study?	
13.	Non-participation	How many people refused to participate or dropped out? What were the reasons for this?	
Setting			
14.	Setting of data collection	Where was the data collected? *E.g. home, clinic, workplace*	
15.	Presence of non-participants	Was anyone else present besides the participants and researchers?	

For peer review only

16.	Description of sample	What are the important characteristics of the sample? *E.g. demographic data, date*	
Data collection			
17.	Interview guide	Were questions, prompts, guides provided by the authors? Was it pilot tested?	
18.	Repeat interviews	Were repeat interviews carried out? If yes, how many?	
19.	Audio/visual recording	Did the research use audio or visual recording to collect the data?	
20.	Field notes	Were field notes made during and/or after the interview or focus group?	
21.	Duration	What was the duration of the interviews or focus group?	
22.	Data saturation	Was data saturation discussed?	
23.	Transcripts returned	Were transcripts returned to participants for comment and/or correction?	
**Domain 3: analysis and findings**			
Data analysis			
24.	Number of data coders	How many data coders coded the data?	
25.	Description of the coding tree	Did authors provide a description of the coding tree?	
26.	Derivation of themes	Were themes identified in advance or derived from the data?	
27.	Software	What software, if applicable, was used to manage the data?	
28.	Participant checking	Did participants provide feedback on the findings?	
Reporting			
29.	Quotations presented	Were participant quotations presented to illustrate the themes / findings? Was each quotation identified? *E.g. Participant number*	
30.	Data and findings consistent	Was there consistency between the data presented and the findings?	
31.	Clarity of major themes	Were major themes clearly presented in the findings?	
32.	Clarity of minor themes	Is there a description of diverse cases or discussion of minor themes?	

When submitting your manuscript via the online submission form, please upload the completed checklist as a Figure/supplementary file.

If you would like this checklist to be included alongside your article, we ask that you upload the completed checklist to an online repository and include the guideline type, name of the repository, DOI and license in the *Data availability* section of your manuscript.

Developed from: Allison Tong, Peter Sainsbury, Jonathan Craig, Consolidated criteria for reporting qualitative research (COREQ): a 32-item checklist for interviews and focus groups, International Journal for Quality in Health Care, Volume 19, Issue 6, December 2007, Pages 349–357, https://doi.org/10.1093/intqhc/mzm042

For peer review only

BMJ Open

Finding the ‘sweet spot’ between customisation and workflows when optimising ePrescribing systems: A multisite qualitative study

Journal:	*BMJ Open*
Manuscript ID	bmjopen-2022-062391.R1
Article Type:	Original research
Date Submitted by the Author:	16-Sep-2022

Complete List of Authors:	Heeney, Catherine; The University of Edinburgh Usher Institute of Population Health Sciences and Informatics<br>Malden, Stephen; The University of Edinburgh Usher Institute of Population Health Sciences and Informatics, Advanced Care Research Centre<br>Sheikh, Aziz; The University of Edinburgh Usher Institute of Population Health Sciences and Informatics, Division of Community Health Sciences

**Primary Subject Heading**:	Health informatics
Secondary Subject Heading:	Qualitative research, Health policy, Health services research
Keywords:	Health informatics < BIOTECHNOLOGY & BIOINFORMATICS, QUALITATIVE RESEARCH, Quality in health care < HEALTH SERVICES ADMINISTRATION & MANAGEMENT, Adverse events < THERAPEUTICS

SCHOLARONE™
Manuscripts

I, the Submitting Author has the right to grant and does grant on behalf of all authors of the Work (as defined in the below author licence), an exclusive licence and/or a non-exclusive licence for contributions from authors who are: i) UK Crown employees; ii) where BMJ has agreed a CC-BY licence shall apply, and/or iii) in accordance with the terms applicable for US Federal Government officers or employees acting as part of their official duties; on a worldwide, perpetual, irrevocable, royalty-free basis to BMJ Publishing Group Ltd ("BMJ") its licensees and where the relevant Journal is co-owned by BMJ to the co-owners of the Journal, to publish the Work in this journal and any other BMJ products and to exploit all rights, as set out in our licence.

The Submitting Author accepts and understands that any supply made under these terms is made by BMJ to the Submitting Author unless you are acting as an employee on behalf of your employer or a postgraduate student of an affiliated institution which is paying any applicable article publishing charge ("APC") for Open Access articles. Where the Submitting Author wishes to make the Work available on an Open Access basis (and intends to pay the relevant APC), the terms of reuse of such Open Access shall be governed by a Creative Commons licence – details of these licences and which Creative Commons licence will apply to this Work are set out in our licence referred to above.

Other than as permitted in any relevant BMJ Author's Self Archiving Policies, I confirm this Work has not been accepted for publication elsewhere, is not being considered for publication elsewhere and does not duplicate material already published. I confirm all authors consent to publication of this Work and authorise the granting of this licence.

For peer review only

Title: Finding the 'sweet spot' between customisation and workflows when optimising ePrescribing systems: A multisite qualitative study

Authors:

Catherine Heeney1, Stephen Malden1,2 (corresponding author), Aziz Sheikh1

Author affiliations:

Corresponding Author email and address:

Dr Stephen Malden

Email: stephen.malden@ed.ac.uk

1. Centre for Medical Informatics

The Usher Institute

The University of Edinburgh

Old Medical School

Teviot Place

Edinburgh

EH8 9AG

2. Usher Institute | Advanced Care Research Centre (ACRC)

Dr Stephen Malden

Usher Institute | Advanced Care Research Centre (ACRC)

University of Edinburgh, Biocubes, Edinburgh BioQuarter

9 Little France Road, Edinburgh, EH16 4UX

Word count: (3909)

Funding: National Institute for Health Research (NIHR) (Optimising ePrescribing in Hospitals (PR-ST-01–10001)/Policy Research Programme).

For peer review only - http://bmjopen.bmj.com/site/about/guidelines.xhtml

Abstract

Objectives: The introduction of ePrescribing systems offers the potential to improve the safety, quality and efficiency of prescribing, medication management decisions and patient care. However, an ePrescribing system will require some customisation and configuration to capture a range of workflows in particular hospital settings. This can be part of an optimisation strategy, which aims at avoiding workarounds that lessen anticipated safety and efficiency benefits. This paper aims to identify ePrescribing optimisation strategies that can be translated into hospitals in different national settings. We will explore the views of professionals of the impact of configuration and customisation on workflow.

Design: This paper draws on 54 qualitative interviews with clinicians, pharmacists and informatics professionals with experience of optimising ePrescribing systems in eight hospital sites and one health system, in four different countries. Interview transcripts were analysed using an inductive thematic analysis.

Setting: Secondary and tertiary care hospitals in the United Kingdom, United States and mainland Europe.

Participants: Fifty-four healthcare workers with expertise in clinical informatics.

Results: Five identified themes following thematic analysis showed that optimisation of ePrescribing systems can involve configuration and/or customisation. This can be a strategy to combat workarounds and to respond to local policy, safety protocols and workflows for particular patient populations. However, it can result in sites taking on responsibility for training and missing out on vendor updates. Working closely with vendors and other users can mitigate the need for extensive system modification and produce better outcomes.

Conclusions: Modifying an ePrescribing system remains key to enhance patient safety and better capture workflow remains key to optimisation. However, we found evidence of an increasingly cautious approach to both customisation and configuration amongst system users. This has led to users seeking to make less changes to the system.

'Strengths and limitations of this study'

- The sample included in this study is comprised of healthcare workers from institutions with extensive experience in the customisation of ePrescribing systems.
- The study sampled from hospitals across different international locations, accounting for differences in policy contexts.
- We focus only on OECD countries, this means that we will have failed to capture interesting examples of ePrescribing beyond that.
- Some types of system are over represented, in particular integrated systems provided by EPIC and Cerner were present in the majority of our sites.

For peer review only

Introduction

Around ten per cent of preventable harms to inpatients is attributable to errors in the prescribing process (1). Electronic health record (EHR) systems offer the potential to improve the safety of prescribing and medication management decisions and patient care (2). The introduction of such systems have the potential to minimise medication error (3). However, it is also acknowledged that an EHR will not be perfectly adapted to capture existing workflows in a given hospital and will require fine tuning to local safety protocols as well as professional and specialisation needs (4, 5). Developing the ePrescribing system in situ is key to making it safe to use, in a 'high risk' setting such as health care (6). In the complex and evolving secondary care context it is unrealistic to imagine that commercial off-the-shelf (COTS) systems can offer optimal performance with local or indeed wider organisational requirements immediately post-implementation (7, 8).

Changes to an ePrescribing system can take the form of configuration and customisation. Configuration works with existing options available in the system or from the vendor by changing and refining rules to reflect processes and practices in local and national settings (7). Customisation involves more fundamental coding changes or 'modifying the underlying software to improve functionality' (9). Hospitals resort to this where the vendor does not provide sufficient configurability to capture necessary workflows, or where it is more financially viable to configure/customise existing systems than it is to purchase additional systems (10).

Workarounds arise where there is a mismatch between practices within particular hospitals and the functionalities and capacities of the ePrescribing system [5(4, 11)] (12-14). They remain a problem especially when they by pass in built safety features [6[7(1)]. Configuration and customisation may

For peer review only
For peer review only - http://bmjopen.bmj.com/site/about/guidelines.xhtml

look like an obvious win in terms of better capturing workflow. Moreover, this route can be necessary where, for example, there are particular patient populations or national policy requirements (10). However, drawbacks of extensive configuration and customisation include being left behind for standard updates from the vendor and uncertainty as to where responsibility for making improvements and routine maintenance resides after customisation of a system has taken place (9, 10).

Modifications to reflect local requirements and system capabilities are key to system optimisation (4, 7, 15), which has been defined as, 'the activity of enhancing system capabilities and integration of subsystem elements to the extent that all components operate at or above user expectations' (16). Hospitals face an inevitable tension between an objective to offer a universal, coordinated and standardised approach to system functionality on the part of the vendor and national health systems, and a requirement to accommodate specific workflows at local level [3, 5(10)]. The impact and learning arising from optimisation strategies, reliant on configuration and customisation, over time is still relatively unexplored in existing literature on ePrescribing in comparison to the equivalent literature on the implementation of new ePrescribing systems to replace paper-based prescribing. Below, we will discuss strategies used to incorporate and manage ePrescribing systems and workflow and the mutual process of change that this interaction produces as well as shifts to in attitudes to modifying system functionality.

Methodology

The qualitative fieldwork described in this paper is part of a wider study on Optimisation of ePrescribing in Hospitals (eP Opt). The methods employed for this present study are outlined below, for a more detailed description see (17). We employed a qualitative multi-site study design with

For peer review only - http://bmjopen.bmj.com/site/about/guidelines.xhtml

semi-structured interviews carried out in each of the study sites. The aim was to capture strategies and practices for optimisation of ePrescribing in hospitals, which are applicable both within the U.K and internationally. .

Selection of study sites:

Eight hospitals and one health care provider participated in this study across four countries (US, UK, Netherlands and Norway). All sites had significant experience of implementation and optimisation of EHRs and ePrescribing, Computerised Physician Order Entry (CPOE) and Clinical Decision Support (CDS). All hospitals had begun digitisation at some level at least a decade before fieldwork began. We selected sites hospitals awarded Healthcare Information and Management Systems Society (HIMSS) level 6 or 7, HIMMS is a widely used measure of digital excellence. The eight hospitals were high profile teaching hospitals. A purposive sampling strategy was used to select cases (18) (see (Supplementary Table 1). We identified sites through a scoping review of optimisation strategies in ePrescribing (19) and two expert roundtable events (17). Only Organisation for Economic Cooperation and Development (OECD) were included to increase the internal comparability of the sample.

(Supplementary table 1)

Data collection:

We contacted 10 hospitals in total. In all cases, we had an initial meeting with a gatekeeper(s) who helped identify relevant professionals who had been expensively involved in their respective

For peer review only - http://bmjopen.bmj.com/site/about/guidelines.xhtml

ePrescribing system optimisations. Due to COVID restrictions and lack of staff availability one site in Spain, did not take part. In the remaining sites (see supplementary Table 1), we contacted relevant professionals by email, with a consent form and information sheet. If participants were willing to participate, they retuned a signed and dated consent form via email to the corresponding researcher(s), who then arranged with them an appropriate time to conduct the interview. We interviewed 54 professionals including clinicians, Chief Information Officers (CIOs), Chief Medical Information Officers (CMIOs), pharmacists and I.T and data specialists [7].

Planned site visits were replaced by remote interviews using approved online platforms, including Teams, Zoom, nhn.no and Skype. Initial contact with the sites was in early 2020, with interviews beginning in the first site in May 2020 and the final interview was conducted in May 2021. Two experienced qualitative researchers (CH and SM) conducted semi-structured interviews following an interview topic guide. The topic guide was designed to investigate the wider ePrescribing optimisations undertaken at the study sites, but included specific questions relating to customisation and configuration. Specifically, participants were asked to detail any customisations/configuration that had taken place at their sites, and to summarise the reasons for this, and the perceived benefits/repercussions of such optimisations on the overall functionality of the ePrescribing systems. Interview questions were developed prior to data collection during "expert roundtable" consultations which involved a structured workshop event. Relevant researchers, policy makers and practitioners in the field of clinical informatics were invited to this event, to help guide the direction of the wider eP Opt project (17). Following the event, the research team developed interview topic guides based on the direction of attending experts, and piloted the questions internally. The researchers had no prior relationship with participants. Interviews lasted between 30 and 90 minutes depending on the interviewees' time and availability and were recorded and transcribed verbatim.

For peer review only - http://bmjopen.bmj.com/site/about/guidelines.xhtml

Data analysis

The research team (CH and SM) first independently coded two transcripts and discussed any discrepancies before finalising the coding framework (supplementary material). The researchers then coded all 54 transcripts. Transcripts were analysed using an inductive thematic analysis and the data grouped into themes and sub-themes (20). We employed NVivo 12 pro qualitative data analysis software. For this paper, we extracted data coded to relevant codes including configuration and workflow, which were then further categorised into five cross cutting themes.

Patient and public involvement

A major component of the eP Opt project is the involvement of patient and public representatives across the four project phases. Specifically, two patient and public (PPI) representatives are involved as team members, who attend research meetings and public events to provide feedback and suggestions on the work within each phase from a patient's perspective. They have also been extensively involved with assisting with the design of an upcoming PPI roundtable event for the project, progress of the study has been shared with a group of invited patients, and their feedback brought to bear on research decisions and directions. These PPI consultations have helped in the formulation of research questions and fed into analysis of the data by highlighting current gaps in practice from a patient perspective. No members of the PPI representatives or wider PPI roundtable group were interviewed as participants in the present study described here.

Results

For peer review only - http://bmjopen.bmj.com/site/about/guidelines.xhtml

Drawing on 54 semi-structured interviews across nine different sites, in four countries (see supplementary Table 2). We identified five themes, influencing both workarounds and configuration: *safety and workarounds, evolution away from highly configurable and customisable solutions, vendor-client relationship, the role of governance* and *finding the 'sweet spot'*. Supporting quotes are provided for each theme, with additional quotes provided in supplementary table 3.

Six of our fieldwork sites had opted to purchase an integrated commercial system, rather than maintaining their own home grown system. Although two sites still employed the so called 'Best of Breed' model (see supplementary Table 2).

(Supplementary table 2.)

Safety and workarounds

As previous work has acknowledged changes to the system are in many cases needed to allow functionality, which enables particular workflows. Changes were needed to reflect workflows beyond the North American context where the system had been developed. Barcode scanning of medicines, which is increasingly a safety feature included in many commercial systems, did not always match the labelling of available products with clear safety, which meant staff created a workaround.

"The biggest problem we have is when we can't scan products. In the beginning, we had a lot of troubles with that because as a nurse I scan the [label] but then I scan the antibiotics and the system says hey, I don't know these antibiotics. So I had to make a workaround …There were a lot of wards who had their own work around, and that's something we discovered in the medication commission

For peer review only

committee, and we had to work at that, and then we discovered it was due to the barcode scanning"- Site C, vendor liaison nurse- Netherlands

Individuals improvised particular processes when they were not sure how to follow the 'standardised route'.

(see quotation 2 *Table 3: supplementary material)*

In some cases, staff struggled to adjust when the system curtailed workarounds. Whilst this increased safety, it meant staff could no longer resort to shortcuts to save time.

(see quotation 3 *Table 3: supplementary material)*

Evolution away from highly configurable and customisable solutions

A number of participants noted the drawbacks of a highly modifiable and flexible systems, whilst accepting the benefits of taking are more cautious approach to modification. Several sites described how vendors had supported a high degree of local customisation or configuration in the early days of implementation. This was often the case when the vendor was trying to roll out their system in a new national or speciality context.

"[COTS 1] very much supported us to, sort of, go out and build things out the way that'd work for us. And a lot of the decisions we made at the very start are things that are coming back and causing

For peer review only - http://bmjopen.bmj.com/site/about/guidelines.xhtml

problems now…is that we went to every single clinic and asked them to design their own documentation. We made a decision that every single clinical trial would have its own set of orders. And so, now we have 4,500 different power plans associated with clinical trials – chemotherapy plans – and it is impossible to maintain"- Site E, CMIO– US.

It later became clear to a number of sites as well as vendors that too much modification could lead to an unwieldy system creating extra work for the vendor.

(see quotation 5 Table 3: Supplementary material)

Whilst the system now has the functionality to mimic the 'traditional drug chart view', this was considered less valuable than the speed and usability for all clinical areas, to which users had become accustomed. Those with responsibility for modifying the system need to balance the safety risks arising from a lack of system functionality with potential over customisation. Experience of the specific needs of a particular site coupled with familiarity with a vendor mean that staff are able to make more informed judgements about whether taking a configuration route was the best way to improve safety overall.

(see quotation 6 Table 3: supplementary material)

Vendor-client relationship

For peer review only - http://bmjopen.bmj.com/site/about/guidelines.xhtml

Several interviewees noted that making highly specific modifications of the system risk opening a gulf between the site and the vendor. Whilst there is a view that sites are clients and have some rights to ask for what they want, a number of interviewees acknowledged that suppliers provide a service at a general level and that the sites can feed into that.

"I talk about influencing the shape of a product it doesn't necessarily mean that you can ask for a very specific bit of functionality that nobody else wants and you're going to get. But actually, as I say, you do have influence if you're doing interesting things that actually the broader health community are interested in. So you've got to constantly have a mind of the suppliers are out there to run their business and the thing that they're really interested in is maximising the reach of their product. So they really want to talk to you when you're doing things that are actually interesting in a broader context…if you drive customisation to a point where what you're doing is unique, you're setting yourself up to fail."- Site A, CIO – UK.

Tailoring the system to a very high degree can also lead to individual sites having to take responsibility for ensuring that staff understand and can safely access the specific customised or configured functionalities.

(see quotation 8 Table 3: supplementary material)

A mutual shaping of needs and vision was occurring in a number of sites, where key individuals had developed close working relationships with the vendors. Those staff selected to complete extensive training pre-implementation would then move on to be 'super users'. This was a mechanism was

For peer review only - http://bmjopen.bmj.com/site/about/guidelines.xhtml

used as a strategy to bring existing workflows together with the system's capacities to avoid both workarounds and excessive customisation.

(see quotation 9 Table 3: supplementary material)

The role of governance

Many interviewees balanced a recognition that changes should be minimal with an acceptance that vendors cannot design systems to fit every context. This meant that governance and monitoring formed a huge part of how staff prioritised and retained particular configurations or customisations.

"So, it's important to have a structure, right. On the pharmacy side, we have a few different committees. We have an adult clinical committee, we have a paediatric committee, we have an oncology committee. So, any drug that we want to configure or optimise or modify really needs to be presented to this committee for ultimate approval. And we have a higher- level governance too."- Site H, pharmacy manager– US.

Internal governance is necessary to consider both petitions for customisation before they are forwarded to the vendor and to prioritise configurations in relation to, for example, alert functions or protocols for specific processes. A data led approach, enabled by system functionality, can be used to inform meetings with relevant professionals regarding prioritisation of particular configurations of the system.

(see quotation 11 Table 3: supplementary material)

For peer review only - http://bmjopen.bmj.com/site/about/guidelines.xhtml

An interviewee with oversight of a number different hospital sites run by a single health provider, monitored and reversed configurations to, for example alert systems, in order to enforce and support uniform expectations about safety functions.

(see quotation 12 Table 3: supplementary material)

Simultaneously, data on any alert rule changes in local hospital sites was reviewed as a basis for prioritising those changes, which were useful at the local level. Staff also need to work towards an awareness that the system at any given time will have limitations and may not be doing everything that people think it does.

(see quotation 13 Table 3: supplementary material)

Finding the 'sweet spot'

Vendors can encourage greater conformity by ensuring that only technologies and practices that follow the rules can take advantage of the interoperability and integration opportunities offered by their products. One US based CMIO made a comparison to the technology company Apple, which limits how its products are modified or used with other products.

For peer review only - http://bmjopen.bmj.com/site/about/guidelines.xhtml

For peer review only

“Apple will say, no, you have to follow our rules, and if your app doesn’t follow these rules, we won’t let you use it on our platform. The overall user experience is tighter and more consistent. I think, [COTS 2] is a bit like that...”- Site H, CMIO– US

In this case, the interviewee did not see the imposition of standards as negative but rather as contributing to a more ‘consistent’ experience for users.

(see quotation 15 Table 3: supplementary material)

One strategy, explicitly encouraged by vendors in some cases, was for individual sites to have their needs met as part of a network of users. Similarly, in the following instance, where the site formed part of a larger health care organisation with one shared system, the was an emphasis placed on only pushing for those changes that could be enacted in every site.

(see quotation 16 Table 3: supplementary material)

The ability to manage the tension between local and wider applicability of a system modification, was referred to as finding ‘a sweet spot’ between ‘out of the box functionality and configuring it.’

Discussion

For peer review only - http://bmjopen.bmj.com/site/about/guidelines.xhtml

This qualitative study involving digitally advanced, hospitals highlights a number of important principles around customisation and configuration to be considered when optimising an ePrescribing system. Misalignment of functionality and organisational needs is seen as one reason why hospitals go down the customisation route (4). Our data suggests that EPIC and Cerner, which were designed in a North American context required changes to functionality to capture workflows in other national settings. The differences in roles, workflows and policy imperatives for access to particular data sources in the different countries required an early rethink soon after implementation. Where sites dealt directly with the vendor, the hospital worked with the vendor to explain and introduce particular national requirements.

Workarounds continue to be a complex issue that require substantial consultation with providers. Workarounds may allow staff and systems to gradually coalesce [7] whilst avoiding the pitfalls of over customisation [19]. We found cautious approach to configuration and customisation was accompanied by low tolerance for the long term use of workarounds. There was a high degree of awareness about the potential safety and administrative drawbacks of by passing the system. Staff had also developed vigilance with regard to the limitations of the system and they need for caution about what safety functions are actually enabled at any given time.

The mismatch between workflow and system functionality has been combatted in a number of sites by so called super users who are deployed to encourage staff to work within the systems capacities. Sites had learned over time to be more adaptable in accepting the way that the balance between their own and vendors' needs. In some cases, key staff members developed a close relationship with the vendor, allowing them to maintain a current appreciation of the potentialities of the system.

For peer review only - http://bmjopen.bmj.com/site/about/guidelines.xhtml

For peer review only

Innovative forms of monitoring and testing the effectiveness of particular configurations were in evidence, with one site in particular employing data to monitor acceptance and use of particular alerts. This data driven approach was combined with staff oversight and comparison with the uptake of similar alerts across other sites. This allowed rigorous monitoring and prioritisation work to balance beneficial and helpful optimisation against the drawbacks of making multiple changes to system. Drawbacks included increasing responsibility on the sites for training, maintenance and improvement.

Finding the 'sweet spot' appeared to involve a subtle evolution away from a demand for highly configurable and customisable solutions and towards finding solutions that could be up scaled. Sites were apt to search for allies within the national system user network to lobby for or finance requested changes to the system. Therefore, in order to fix a mismatch between functionality and workflow sites would need to be proactive in finding other users experiencing similar problems. There appeared to be an increasing willingness for sites to encourage behaviour change in staff and to scale back their requests for changes to the system driven by both safety and usability concerns.

Strengths and limitations

This is one of the first studies to investigate the impact of ePrescribing optimisation attained by customisation and configuration practices. The case studies presented provide insight into diverse regional and national contexts. The focus on optimisation enables the sharing of key learning in the interactive evolution of users and system. By focussing on professionals within advanced sites, we have been able to capture the benefits of their experiences post implementation.

For peer review only - http://bmjopen.bmj.com/site/about/guidelines.xhtml

Due in part to the disruption caused during fieldwork by COVID-19, we did not collect equal levels of data in all sites. This was mitigated wherever possible by our carrying out longer interviews. Hospitals using two integrated systems or COTS (Epic and Cerner) are dominant within the sample, best-of-breed systems are far less represented. The US and UK contexts are over represented compared to the Netherlands and Norway. The focus on OECD member countries narrowed the range of hospitals we could include.

Interpretation in the light of the wider published literature

Customisation has been presented as giving positive outcomes for users and patients (21). It is also inevitable given the difficulty of capturing workflows without knowledge of the environment and actual challenges faced by staff (8). Whilst the system may be designed to reshape practices in a more efficient and safer ways, initially there is often misalignment between what is required by the specialisation or organisation and the functionality of the system (4, 10). Vendors worked closely with early adopters bringing a lot of specialised knowledge about national settings and specialities to the relationship, which our data suggests enabled customisation. This appeared to become less desirable not only to vendors but more recently also to users, over time. Hospital sites are increasingly willing to adopt a parsimonious approach to configuration and customisation.

The mutual learning between site and vendor is an ongoing process requiring staff and resourcing. Where systems are developed in a distinct national context a network of other users may be sought out in developing necessary functionality at a quicker pace. More recent studies have pointed to greater awareness of the balance of costs and benefits in relation to extensive modifications, including wide variation between sites using the same system (4, 22). Future research could look at

For peer review only - http://bmjopen.bmj.com/site/about/guidelines.xhtml

the ways in which relationships between vendor and individual sites are shaped by the presence of a critical mass of users in a given national context.

Conclusion

Our data suggests an increasing acceptance for interviewees that the needs of individual sites would be met as part of a network of users potentially of the same product. Whilst some frustrations with delays on the part of the vendor to changes required by sites remained, there was not much enthusiasm for making too many changes at a local level. Sites acknowledged the danger of becoming too responsible for an extremely bespoke system. Simultaneously, interviewees were cognisant that the vendor would not foresee all eventualities, especially in specialities or within scientifically as well as digitally advanced hospitals. However, they had learned the benefit of considering broad applicability of optimisations.

Contributors

AS conceptualised the project and designed the study. CH recruited study sites; CH and SM recruited individual participants within study sites and conducted data collection. CH prepared the first draft of the manuscript. All authors contributed to the final draft of the manuscript and approved it for submission.

Competing interests:

None

Ethics approval:

For peer review only - http://bmjopen.bmj.com/site/about/guidelines.xhtml

This study was granted ethical approval by the Usher Research Ethics Group (University of Edinburgh) on 21/01/2020 (ref.1906), and the relevant NHS research and development approvals were acquired for UK-based sites on 23/01/2020 (ref.19/HRA/7015). As this research is exclusively being conducted with healthcare professionals, there is no involvement of vulnerable groups. As we are focusing this research on well-known, world-leading institutions in health informatics, extra care should be taken when presenting research findings to not compromise the anonymity of the respondents. Specific job titles have been masked when used in conjunction with individual quotes, and care has been taken to avoid any quotes that could be used to potentially identify individuals. All audio files, transcripts and consent forms will be kept in a secure, password-protected folder within the University of Edinburgh's DataStore (for further information please consult the protocol for this study (17)).

Acknowledgments:

We thank all the participating sites and the individuals who participated in an interview for this study. We thank Serena Tricarico, Kieran Turner, Toni Wigglesworth, and our Patient and Public Involvement representatives, Antony Chuter and Jillian Beggs, for their support and feedback throughout the project. We also acknowledge the support of colleagues from the Department of Health and Social Care, the National Health Service and the Medicines and Healthcare products Regulatory Agency: Ann Slee (NHS), Jason Cox (DHSC), Richard Cattell (NHS), Helen Causley (DHSC), Paul Stonebrook (DHSC), Mick Foy (MHRA), Kathryn Ord (MHRA), and Graeme Kirkpatrick (NHS). We thank the referees for reviewing this manuscript.

Data availability statement:

Data are available from the corresponding author upon reasonable request.

Funding:

For peer review only - http://bmjopen.bmj.com/site/about/guidelines.xhtml

This study/project is funded by the National Institute for Health Research (NIHR) (Optimising ePrescribing in Hospitals (PR-ST-01–10001)/Policy Research Programme). The views expressed are those of the author(s) and not necessarily those of the NIHR or the Department of Health and Social Care.

For peer review only

For peer review only - http://bmjopen.bmj.com/site/about/guidelines.xhtml

References

1. Puaar SJ, Franklin BD. Impact of an inpatient electronic prescribing system on prescribing error causation: a qualitative evaluation in an English hospital. BMJ Qual Saf. 2018;27(7):529-38.
2. Cornford T, Dean B, Savage I. et al. Electronic prescribing in hospitals-challenges and lessons learned. 2009.
3. Bates DW, Teich JM, Lee J. et al. The impact of computerized physician order entry on medication error prevention. J Am Med Inform Assoc. 1999;6(4):313-21.
4. Wiegel V, King A, Mozaffar H. et al. A systematic analysis of the optimization of computerized physician order entry and clinical decision support systems: A qualitative study in English hospitals. Health Informatics J. 2019:1460458219868650.
5. Salmon JW, Jiang R. E-prescribing: history, issues, and potentials. Online J Public Health Inform. 2012;4(3).
6. Wachter R. Making IT work: harnessing the power of health information technology to improve care in England. London, UK: Department of Health. 2016.
7. Anderson S, Hardstone G, Procter R. et al. Down in the (data) base (ment): Supporting configuration in organizational information systems. Resources, co-evolution and artifacts: Springer; 2008. p. 221-53.
8. Coiera E. Designing interactions. ROUTLEDGE HEALTH MANAGEMENT SERIES. 2003:97.
9. Sittig DF, Belmont E, Singh H. Improving the safety of health information technology requires shared responsibility: It is time we all step up. Healthcare; 2018: Elsevier.
10. Mozaffar H, Williams R, Cresswell KM. et al. The challenges of implementing packaged hospital electronic prescribing and medicine administration systems in UK hospitals: premature purchase of immature solutions? Information Infrastructures within European Health Care. 2017:129-49.
11. Mozaffar H, Cresswell KM, Williams R. et al. Exploring the roots of unintended safety threats associated with the introduction of hospital ePrescribing systems and candidate avoidance and/or mitigation strategies: a qualitative study. BMJ Qual Saf. 2017;26(9):722-33.
12. Boonstra A, Jonker TL, van Offenbeek MA et al. Persisting workarounds in Electronic Health Record System use: types, risks and benefits. BMC Med Inform Decis Mak.2021;21(1):1-14.
13. Cresswell KM, Mozaffar H, Lee L et al. Workarounds to hospital electronic prescribing systems: a qualitative study in English hospitals. BMJ Qual Saf. 2017;26(7):542-51.
14. Ser G, Robertson A, Sheikh A. A qualitative exploration of workarounds related to the implementation of national electronic health records in early adopter mental health hospitals. PloS One. 2014;9(1):e77669.
15. Moon MC, Hills R, Demiris G. Understanding optimisation processes of electronic health records (EHRs) in select leading hospitals: a qualitative study. BMJ Health Care Inform. 2018;25(2).
16. Kidd TT. Handbook of research on technology project management, planning, and operations: IGI Global; 2009.
17. Heeney C, Malden S, Sheikh A. Protocol for a qualitative study to identify strategies to optimise hospital ePrescribing systems. BMJ Open. 2021;11(1):e044622.
18. Patton MQ. Qualitative research & evaluation methods: Integrating theory and practice: Sage publications; 2014.

For peer review only - http://bmjopen.bmj.com/site/about/guidelines.xhtml

19. Williams J, Malden S, Heeney C. et al. Optimizing Hospital Electronic Prescribing Systems: A Systematic Scoping Review. Journal of Patient Safety. 2021.
20. Braun V, Clarke V. Using thematic analysis in psychology. Qualitative research in psychology. 2006;3(2):77-101.
21. Adeola M, Azad R, Kassie GM. et al. Multicomponent interventions reduce high-risk medications for delirium in hospitalized older adults. Journal of the American Geriatrics Society. 2018;66(8):1638-45.
22. Pontefract SK, Hodson J, Slee A. et al. Impact of a commercial order entry system on prescribing errors amenable to computerised decision support in the hospital setting: a prospective pre-post study. BMJ Qual Saf. 2018;27(9):725-36.

For peer review only

For peer review only - http://bmjopen.bmj.com/site/about/guidelines.xhtml

Supplementary tables

Supplementary Table 1. Site Selection Criteria

Criterion	Examples
**Significant post implementation experience of ePrescribing system**	HIMMS/Digitally mature (years since EHR implementation)
**Available points of comparison for health system to NHS**	OECD country
**EHR system**	Large integrated systems and Best of Breed
**Vendor**	A mixture of home grown and commercial off the shelf package providers
**Innovative approach**	For example, integrating genomics and other biomedical data, big data feedback into ePrescribing
**Prior interaction with the site/named contact?**	Gatekeeper/ network

For peer review only

Supplementary Table 2. eP Opt Study site characteristics

	Hospital details			Participant details			
Site identifier	Location	Size	Type	Roles included in sample	Total number	Vendor or home-grown	Integrated or best of breed (BoB)
Site 1	UK	~760 beds	Teaching hospital	Pharmacy managers, analysts, pharmacists, nurses, information officers	6	Vendor	BoB

Site 2	UK	~800 beds	Teaching hospital	Pharmacy managers, physicians, analysts, pharmacist,<br><br>Nurses,<br><br>Other Ancillary care	13	Vendor	Integrated
Site 3	Netherlands	953 beds	Teaching hospital	Clinical pharmacist, nurses, Chief clinical information officer	5	Vendor	Integrated
Site 4	Norway	1,870 beds	Teaching hospital	Pharmacy, physician, nurse, central health I.T clinician	5		
Site 5	US	~80 beds	Paediatric Cancer hospital	Pharmacy managers, physicians, analysts, information officers	9	Vendor	Integrated
Site 6	US	~800 beds	Teaching hospital	Pharmacy managers, physicians, analysts, pharmacists	8	Vendor	Integrated
Site 7	US	~670 beds	Teaching hospital	Physicians,<br><br>nurses	3	Home-grown	BoB
Site 8	US	~1500 beds	Teaching hospital	Pharmacy managers, physicians, pharmacists<br><br>Information officers	5	Vendor	Integrated
Site 9	US	~20,000 *	Healthcare Provider	Informatics and	2	Home grown	Integrated

For peer review only

				pharmacy leads			

Supplementary table 3. Quotations by theme.

Quotation number in main manuscript	Interviewee Pseudonym	Quotation
**Safety and workarounds**		
Quotation 2	Site B, pharmacist safety– UK	"Even though they've been taught a standardised route early on they don't remember that. And if you find another way you can find what they believe is a shortcut to do something, but often then some of the safety functions aren't on it because they've gone in a different way."
Quotation 3	Site C, CMIO – Netherlands	*"I mean when I was a CMIO one of the nurses from the gynaecology department, they came to me and they said, hey, we have a problem with Epic, we cannot prescribe medication any more for our ambulatory patients. I said, well, you're not allowed to, legally you're not allowed to prescribe. Yes, but we always did. Yeah, okay, but that was against the law then. Yes, but how can we work that? I said, and how did you do it then, because you had to sign for it? Oh, yes, but we simply always had a blank book of signed prescriptions, they were blank and we just filled in what was needed."*
**Evolution away from highly configurable and customisable solutions**		
Quotation 5	Site F, ambulatory care doctor– US	*"Lot of what we have is customised to us. Some would argue that it's over-customised in that over-customisation you make things so complex that it's hard to like…they become very difficult to work with and very cumbersome".*
Quotation 6	Site E, CDS officer– US	*"Then, it usually gets reviewed and if it's the medication safety group especially, and if it's something we feel like might repeat itself and the rules system can't handle it then we*

For peer review only - http://bmjopen.bmj.com/site/about/guidelines.xhtml

		take it through that process of, should we do this, should we put the effort into doing that. And, we might respond and create a custom decision support rule to take care of it, if we feel like it might never happen again, we might not or it's just so complicated that you can't prevent it..“
**Vendor-client relationship**		
Quotation 8	Site B, division pharmacist– UK	"I suppose it depends as well a lot of the functionality we can develop, so the [COTS 1] we've got is in lots of other sites. It's different in all of the sites because people tailor it to what they believe are their own needs... And then if you tailor it so much then you need to be able to deliver all, you almost take more responsibility for your training and everything."
Quotation 9	Site D, IT nurse- Norway	" I did work with the doctors in that part of the project, so we did go around, talk to the doctors, what do they need to learn more about, and what was the frustration, and that we work around that and make new syst…learned how to work smarter for the doctor. So I did go out on the wards and talk to the doctors in their meetings."
**The role of governance**		
Quotation 10	Site H, pharmacy manager– US	*"So, it's important to have a structure, right. On the pharmacy side, we have a few different committees. We have an adult clinical committee, we have a paediatric committee, we have an oncology committee. So, any drug that we want to configure or optimise or modify really needs to be presented to this committee for ultimate approval. And we have a higher- level governance too."*
Quotation 11	Site E, CDS officer– US	*"…the …medication safety resident [has] taken that governance to a little bit of a higher level. Where, he's developed a group of physicians, advanced practice providers, pharmacists, IT professionals that review all those*

For peer review only

		decisions/rules that we have that affect medications and get them to prioritise things. Also, puts data in front of them in how often things are firing, asks for voting on whether or not, you know, a specific piece of decision support should be turned off or enhanced or whatever"
Quotation 12	Site I, pharmacy informatics– US	*"So, we have the ability to check drugs, allergy interactions, across different facilities, so it's called a remote data order check…. …So, we had, we had quite a few instances where patients had a recorded something up, another drug or drug allergy and there are clinical decisions for it, but the electronic medication ordering did not trigger. Why didn't it? And then we were able to say well, the feature was turned off, and now we actually monitor within a day, so if someone turns that feature off today we would know by tomorrow morning and contact them turning it back on again."*
Quotation 13	*Site B, pharmacist safety– UK*	*"I think our electronic prescribing system was chosen because it links into the results and the patient record, so it's one solution, which is brilliant. But then some of the functionality from a prescribing and administration point of view is not there, so therefore that creates risks that we have to then look at… So, if the doctor then says, do you know what, I am going to give it anyway, it doesn't fire an alert for the nurse as they go to administer it; whereas other systems do do that. So, that is an accepted risk with this system. So, we then have to put other systems in place to make staff aware of that and to support that functionality."*
**Finding the 'sweet spot'**		
Quotation 15	*Site I, pharmacy informatics– US*	*"So they would take a facility's innovation and then distribute it to all of the organisation so that it's no*

For peer review only

		longer a customisation, now it's just a feature."
Quotation 16	*Site A, CIO – UK*	*"So really all the time you're trying to pay for a bit of a sweet spot in terms of taking out of the box functionality and configuring it. Is probably a way of describing it. where you're not fundamentally changing the product, but if you can put in configurations that are informed again from the point of view of what has kind of a broad applicability across your profession, and not too specific, tends to give good results."*

For peer review only

Consolidated criteria for reporting qualitative studies (COREQ): 32-item checklist

Please indicate in which section each item has been reported in your manuscript. If you do not feel an item applies to your manuscript, please enter N/A.

For further information about the COREQ guidelines, please see Tong *et al.*, 2017: https://doi.org/10.1093/intqhc/mzm042

No.	Item	Description	Section #
**Domain 1: Research team and reflexivity**			
Personal characteristics			
1.	Interviewer/facilitator	Which author/s conducted the interview or focus group?	
2.	Credentials	What were the researcher's credentials? *E.g. PhD, MD*	
3.	Occupation	What was their occupation at the time of the study?	
4.	Gender	Was the researcher male or female?	
5.	Experience and training	What experience or training did the researcher have?	
Relationship with participants			
6.	Relationship established	Was a relationship established prior to study commencement?	
7.	Participant knowledge of the interviewer	What did the participants know about the researcher? *E.g. Personal goals, reasons for doing the research*	
8.	Interviewer characteristics	What characteristics were reported about the interviewer/facilitator? *E.g. Bias, assumptions, reasons and interests in the research topic*	
**Domain 2: Study design**			
Theoretical framework			
9.	Methodological orientation and theory	What methodological orientation was stated to underpin the study? *E.g. grounded theory, discourse analysis, ethnography, phenomenology, content analysis*	
Participant selection			
10.	Sampling	How were participants selected? *E.g. purposive, convenience, consecutive, snowball*	
11.	Method of approach	How were participants approached? *E.g. face-to-face, telephone, mail, email*	
12.	Sample size	How many participants were in the study?	
13.	Non-participation	How many people refused to participate or dropped out? What were the reasons for this?	
Setting			
14.	Setting of data collection	Where was the data collected? *E.g. home, clinic, workplace*	
15.	Presence of non-participants	Was anyone else present besides the participants and researchers?	

For peer review only

16.	Description of sample	What are the important characteristics of the sample? *E.g. demographic data, date*	
Data collection			
17.	Interview guide	Were questions, prompts, guides provided by the authors? Was it pilot tested?	
18.	Repeat interviews	Were repeat interviews carried out? If yes, how many?	
19.	Audio/visual recording	Did the research use audio or visual recording to collect the data?	
20.	Field notes	Were field notes made during and/or after the interview or focus group?	
21.	Duration	What was the duration of the interviews or focus group?	
22.	Data saturation	Was data saturation discussed?	
23.	Transcripts returned	Were transcripts returned to participants for comment and/or correction?	
**Domain 3: analysis and findings**			
Data analysis			
24.	Number of data coders	How many data coders coded the data?	
25.	Description of the coding tree	Did authors provide a description of the coding tree?	
26.	Derivation of themes	Were themes identified in advance or derived from the data?	
27.	Software	What software, if applicable, was used to manage the data?	
28.	Participant checking	Did participants provide feedback on the findings?	
Reporting			
29.	Quotations presented	Were participant quotations presented to illustrate the themes / findings? Was each quotation identified? *E.g. Participant number*	
30.	Data and findings consistent	Was there consistency between the data presented and the findings?	
31.	Clarity of major themes	Were major themes clearly presented in the findings?	
32.	Clarity of minor themes	Is there a description of diverse cases or discussion of minor themes?	

When submitting your manuscript via the online submission form, please upload the completed checklist as a Figure/supplementary file.

If you would like this checklist to be included alongside your article, we ask that you upload the completed checklist to an online repository and include the guideline type, name of the repository, DOI and license in the *Data availability* section of your manuscript.

Developed from: Allison Tong, Peter Sainsbury, Jonathan Craig, Consolidated criteria for reporting qualitative research (COREQ): a 32-item checklist for interviews and focus groups, International Journal for Quality in Health Care, Volume 19, Issue 6, December 2007, Pages 349–357, https://doi.org/10.1093/intqhc/mzm042

For peer review only
